# Supplementary material for: The Effect of Ethylene on the Color Change and Resistance to Botrytis cinerea Infection in ‘Kyoho’ Grape Fruits
Source: Foods. 2020 Jul 7;9(7):892. doi: 10.3390/foods9070892 (PMC7404975; doi:10.3390/foods9070892)
Supplement: Supplementary file 1 [file foods-09-00892-s001.pdf]

Table S1. RT-qPCR primer sequences used in this study.

Gene Name	Accession No.	Forward primer sequences (5′→3′)	Reverse primer sequences (5′→3′)
VvACTIN	XM_002282480	GATTCTGGTGATGGTGTGAGT	GACAATTTCCCGTTCAGCAGT
VvUFGT	AF000372	GGGATGGTAATGGCTGTGG	ACATGGGTGGAGAGTGAGTT
VvGST	AY971515	ACTTGGTGAAGGAAGCTGGA	TTGGAAAGGTGCATACATGG
VvCHS	AB015872	AAACTATGTGCTACAGTCC	GACTACAGTTCAGAAATAA
VvCHI	X75963	CAGGCAACTCCAT TCTTTTC	TTCTCTATGACTGCATTCCC
VvF3H	X75965	CCAATCATAGCAGACTGT CC	TCAGAGGATACACGGTTGCC
VvMYBA1	KY406228.1	TAGTCACCACTTCAAAAAGG	GAATGTGTTTGGGGTTTATC
VvMYB2	AB097924	CGAGCAGGGTTGAATAGATG	CTACCCGCAATCAAGGAC
VvF3′H	XM_002284115.4	AAAACCTACGGCCCTCTCAT	AGGAGGCCTGTTGGAGAAAT
VvF3′5′H	AB213606	AAACCGCTCAGACCAAAACC	ACTAAGCCACAGGAAACTAA
VvPME	NM_001281162	TGGTGAAGAAGGGCTTACC	CATAGTAGAGTGTATCCAGAGC
VvPL	XM_002285603	TCCAACAACCACTTTGCC	CACTTCCACCAATAGCATAC
VvPG	XM_002281791	CTTTGAGCCTTTGATACAGC	CCTGAACACCATCTCCTTTC
VvCell	XM_010661836	GGCTAAGAAGCAGGTTGAC	CAGAGTAGAGGTATTGGAAGC
VvQR	XM_003631207	GTGATAAGGCAGTGAAGGTA	CCTCTCCGTTTGAAGTTACT
VvEGS	XM_003631651	AGTATTACCGTCTATGGCAG	TTCACAACCTCTTCCTCTG-
VvECar	JF808010	ATGGCTATACTC ATTCTGGGAC	ATCTCTCCAAGCAAGCAGCA
VvNCED1	AY337613.1	CGGAGAGGTTCGTAGTGGTT	CAGAGATGGAAGCAGAAGCAAT
VvNCED2	AY337614.1	TTCGCCATCACAGAGAATTACG	CAGAGGTGGAAGCAGAAGGT
VvNCED3	XM_002283149	GTAGGCTTGGTGGATGGAT	TCGTGGATAACAGGTGAGC
VvBG1	GU480917	TGAACCTTACATAGTTGCCCACCAT	AATCCCCATACATCAGAGGGTCAAT
VvBG2	XM_003632325	ATAGTGAAGAAGAGGGCAGGCACG	GCGGCCATATCTGCAAGAAAGTC
VvBG3	XM_002272377	GCCGCAGAATAGTAGAAGACTTTGC	GCAATATAAGGCTCGGTTGATGAGT-
VvCYP707A1	XM_002282197	GGTCACTTGGAGGGTAATTAC	TGTTGTCGGCGATTTGATCCT
VvTAA1	XM_002281372	GCTAAAGTGTGAAGGAGGC	CTGAGATGGAGACTGGACA
VvINS	XM_002281563	TGGTGTGATAGTTGGTAGTG	AGACTTCAGGGATTTGGTG
VvYUCCA	XM_002280979	AGAGAAAGATGGGTTGCCC	AAAGAAGCACCAAGCAGG
VvPIN1	XM_002284266	TGGAATGGCTATGTTCAGC	GACGAGGGTAATCGGTAAC
VvLOX	XM_010661517	ACACATAGAGAATCACCTGG	TGGCTTCAAAGTTCCGTC
VvAOS	XM_002283744	GACCTGAAACCGAGAATCC	CAAAGGAATCGTAACGCAG
VvCOI1	XM_002276109	TGAGGTATTTGTGGGTGC	TCCAGCAAGTGAGTAGTATG
VvJAZ4	XM_002272327.4	TTCAGGAAATCGGCAACAACAGA	CCCTTGGCGGCTAATAGCATG
VvJAZ9	XM_002277121.3	TTTACCGGGCAGAGAGCGCC	GATTCGGGCGTGCCGTTTCC
VvACS1	XM_002278453.4	CTCAGGTAATTGTCGGATGTG	GATCAAAACGAGAATCCGGT
VvACO2	XM_010648823.2	CTGTTGTCGTTCTAGTGTTGGTA	CATCAAACCATACCTTAGGAGCT
VvETR2	XM_002284471.3	TTTGCACCAAAAGCATGGCTC	GGTTCAGAAATGTTGATTCC
VvEIN3	XM_010660723.2	CGAGCACAGGATGGGATACT	TCCGATCAAACCTCACCTTC
VvDWF1	XM_010656104.2	ACCGAGAAGGAAGTGCAGGAG	ACCATCACATTCGTTGAGCAGG
VvBR6OX1	NM_001280960.1	GACAAGAGCTTAGAGTCCCAAAAC	GAAAATTATTGTACATCCATATTGCTT
VvAPX	XM_010655137.2	TGATGCTTCGACTAGCATGG	AGGATCGGAAATTGCTCCTT
VvPAD4	XM_010654614.2	GGAAAGCTTCTTGGCAGTTG	TTGGCCATCTTTAGGCAATC
VvNPR1	XM_002281439	GTGTTTTTCAAGGCCGTGTT	AGCACCTCCACCATGAAATC
VvWRKY 70	XM_002272468.3	GGCGAAAATACGGACAAAAA	TGACTTCAGCATGCTTTTGC
VvMYC2	XM_002280217.4	GATTCACACGCAGGGTTTTT	GGCACTTCCTCTTGAAGTCG
Vvpgip	JN797496.1	CCGGGAAAATCCCATATTCT	AAGGTCCAACGACGTCAAAC
VvDREB	XM_004305310	CTAAGGACATACAGACGGC	TTGTTCCGATTCCGCTTC
VvNAC26	KT216259.1	GCATGCCCCGTATCCATTATCC	CTTGTCTGTCCCTGTGGCTNC
VvPIP1	KJ697715.1	CCAAGAGGAGTGCCAGAGAC	CCAGGCCTTCTCATTGTTGT

Table S2. Primers used for Yeast Two-Hybrid analysis.

Gene name	Primer sequence	Restriction Enzyme cutting site
BD-VvERF1-F	CGGGATCCATGGAAATGGAGTCCTACTC	*BamH*I
BD-VvERF1-R	CCGCTCGAGAGCACCCAAATCCTCTAATAC	*Xho*I
AD-VvEIN3-F	CGGGATCCCTTGAGTTTATGTCCGTTCC	*BamHI*
AD-VvEIN3-R	ACGCGTCGACCCAGATAGAAGCATCCTGC	*Sal*I
3-AD	CTGTGCATCGTGCACCATCT	
3-BD	GACTCTTAGGTTTTAAAACGAAAA	
T7	TAATACGACTCACTATAGGGCG	
